# The Gut–Brain Axis and Its Relation to Parkinson’s Disease: A Review

**DOI:** 10.3389/fnagi.2021.782082

**Published:** 2022-01-07

**Authors:** Emily M. Klann, Upuli Dissanayake, Anjela Gurrala, Matthew Farrer, Aparna Wagle Shukla, Adolfo Ramirez-Zamora, Volker Mai, Vinata Vedam-Mai

**Affiliations:** ^1^Department of Epidemiology, College of Public Health and Health Professions & College of Medicine, University of Florida, Gainesville, FL, United States; ^2^Emerging Pathogens Institute, University of Florida, Gainesville, FL, United States; ^3^Department of Neurology, College of Medicine, University of Florida, Gainesville, FL, United States; ^4^Norman Fixel Institute for Neurological Diseases, University of Florida, Gainesville, FL, United States

**Keywords:** Parkinson’s disease, gut–brain axis, microbiome, alpha-synuclein, dysbiosis

## Abstract

Parkinson’s disease is a chronic neurodegenerative disease characterized by the accumulation of misfolded alpha-synuclein protein (Lewy bodies) in dopaminergic neurons of the *substantia nigra* and other related circuitry, which contribute to the development of both motor (bradykinesia, tremors, stiffness, abnormal gait) and non-motor symptoms (gastrointestinal issues, urinogenital complications, olfaction dysfunction, cognitive impairment). Despite tremendous progress in the field, the exact pathways and mechanisms responsible for the initiation and progression of this disease remain unclear. However, recent research suggests a potential relationship between the commensal gut bacteria and the brain capable of influencing neurodevelopment, brain function and health. This bidirectional communication is often referred to as the microbiome–gut–brain axis. Accumulating evidence suggests that the onset of non-motor symptoms, such as gastrointestinal manifestations, often precede the onset of motor symptoms and disease diagnosis, lending support to the potential role that the microbiome–gut–brain axis might play in the underlying pathological mechanisms of Parkinson’s disease. This review will provide an overview of and critically discuss the current knowledge of the relationship between the gut microbiota and Parkinson’s disease. We will discuss the role of α-synuclein in non-motor disease pathology, proposed pathways constituting the connection between the gut microbiome and the brain, existing evidence related to pre- and probiotic interventions. Finally, we will highlight the potential opportunity for the development of novel preventative measures and therapeutic options that could target the microbiome–gut–brain axis in the context of Parkinson’s disease.

## Introduction

**FIGURE 1:**
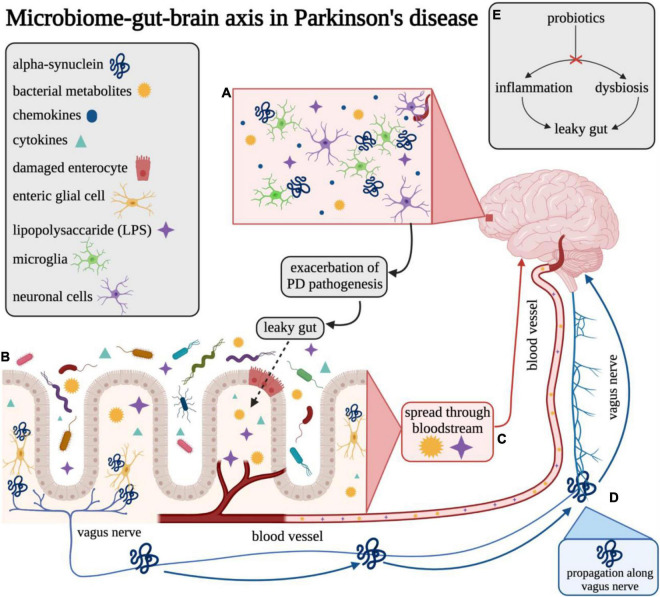
Overview of the gut–brain axis in Parkinson’s disease. Visual overview of the proposed microbiome–gut–brain axis in Parkinson’s disease. **(A)** LPS and other bacterial metabolites may be able to enter the brain across the blood–brain barrier (BBB) and may elicit release of various chemokines/cytokines that promote an inflammatory response in Parkinson’s disease. **(B)** Microbes in the gut lumen can promote inflammatory pathways and cause damage to enterocytes which may lead to compromised gut epithelial barrier integrity (“leaky gut”). **(C)** Bacterial metabolites, such as LPS, can translocate from the gut lumen to the bloodstream across the compromised gut barrier and cause possible systemic and neuroinflammation in the brain. **(D)** Misfolded α-synuclein may be induced by microbes at the intersection of the gut lumen and ENS and may be propagated to neurons in the brain through the vagus nerve. **(E)** Probiotic interventions are thought to reverse dysbiosis through altering the composition of the microbiome. This change is also purported to result in reduction of inflammation and improvement of gut epithelial barrier integrity, thereby preventing or reducing microbial translocation. This figure was created with BioRender.com.

Parkinson’s disease (PD) is the second most common neurodegenerative disorder with an estimated prevalence of approximately 1% among individuals over the age of 65 (Nussbaum, 2003). The worldwide burden of PD has more than doubled over the past three decades, increasing from 2.5 million patients in 1990 to over 6.1 million in 2016 (GBD 2016 Parkinson’s Disease Collaborators, 2016; Rocca, 2018). This trend is expected to continue in the coming generations as the global population continues to increase in age. In addition, period effects also suggest the age-adjusted incidence of PD is increasing among males (Rocca, 2018). Hence, the prevalence of Parkinson’s Disease (PD) is estimated to reach nearly 1,238,000 cases by the year 2030 in the United States alone (Marras et al., 2018). PD is a progressive neurodegenerative disease characterized by the pathological misfolding of alpha-synuclein (α-syn) protein which subsequently impacts the function of the central, peripheral, and enteric nervous systems (Kouli et al., 2018). PD is also a multifactorial disease with both genetic (e.g., 23 genes are linked to Mendelian forms of parkinsonism, and 187 genes in ∼90 loci have been associated with idiopathic PD) and environmental (e.g., head injury, cigarette smoking, caffeine consumption, exposure to certain pesticides/herbicides or heavy metals, etc.) risk factors (Kouli et al., 2018). Misfolded α-syn aggregates, otherwise known as Lewy body deposits, contribute to the degeneration of dopaminergic neurons within the *substantia nigra* and other related circuitry (Spillantini et al., 1997, 1998; Trojanowski and Lee, 1998), and ultimately the onset of several cardinal motor and non-motor features of PD including dementia, gastrointestinal (GI) dysfunction, tremor, postural instability, bradykinesia, and rigidity (Váradi, 2020). Although most commonly located in the brain, α-syn aggregates have also been found in peripheral locations, such as the enteric nervous system (ENS), lending support to the idea of a “gut–brain axis,” a bidirectional communication pathway between the central and enteric nervous systems and the GI system (Chao et al., 2020; Figure 1).

## Alpha-Synuclein Pathology in Parkinson’s Disease

Alpha-synuclein is a neuronal protein found at synapses and is abundantly found in the brain (Figure 1A) and is associated with the neuropathology observed in PD and related neurodegenerative disorders (e.g., Lewy Body Disease and Multiple System Atrophy), predominantly through the formation of aberrant aggregates that may disrupt cellular homeostasis, synaptic function, and induce neuronal degeneration (Spillantini et al., 1997, 1998; Trojanowski and Lee, 1998; Stefanis, 2012). Notably, α-syn is released through an unique secretory pathway (Lee et al., 2005, 2016; Emmanouilidou et al., 2010; Gustafsson et al., 2018), and although the exact mechanisms of fibrillar α-syn cellular secretion and uptake are not well understood, extracellular forms of this protein have been found in both rodent and human brain and interstitial fluid, providing a potential avenue for intercellular propagation (Emmanouilidou et al., 2011). A recent study using primary neuronal cell cultures and *in vivo* microdialysis found that approximately 70% of extracellular α-syn originates from neuronal activity-dependent pathways (e.g., glutamatergic neurotransmission) (Yamada and Iwatsubo, 2018). Further, microglial activation and subsequent neuroinflammation is associated with misfolded α-syn in PD (Alvarez-Erviti et al., 2011; Xia et al., 2019). Whether neuroinflammation triggers protein misfolding or, alternatively, the presence of misfolded α-syn promotes a neuroinflammatory response capable of initiating PD pathology, has not yet been definitively determined and neither is mutually exclusive. While several potential factors associated with α-syn misfolding and aggregation have been identified [e.g., intra- and extra-cellular pH (Buell et al., 2014), ionic concentration (de Oliveira and Silva, 2019), presence of metals (Villar-Piqué et al., 2016)], the underlying mechanisms are still largely unknown. Clearly, chronic and constitutive α-syn gene expression is a major risk factor as gene dose is inversely correlated with age at onset (Book et al., 2018).

## Potential Contributions of Microbiota to Alpha-Synuclein Pathologies: Braak’s Hypothesis

The presence of healthy intestinal microbiota promote the integrity of the blood–brain barrier (BBB) through regulation of tight junction protein expression (e.g., occludin and claudin-5) mediated by short chain fatty acids (SCFAs) (Hoyles et al., 2018; Tran and Mohajeri, 2021). SCFAs play an important role in maintaining intestinal barrier integrity by preventing microbial translocation, that is known to be associated with local intestinal inflammation, systemic inflammation and neuroinflammation (Houser and Tansey, 2017; Wang et al., 2020). However, dysbiosis of the microbiome, associated with an increased abundance of potentially detrimental bacteria, can compromise gut barrier integrity through bacterial production of endotoxins (e.g., lipopolysaccharide) capable of altering immune response, initiating proinflammatory pathways, and directly damaging intestinal epithelial cells (Ghosh et al., 2020). In the bloodstream, lipopolysaccharide (LPS) interacts with immune cells, upregulates systemic expression of proinflammatory cytokines (e.g., TNF and interleukins) and inflammation (Ghosh et al., 2020), and, in high concentrations, may directly disrupt the BBB to induce neuroinflammation (Banks et al., 2015). Further, LPS has been found to induce a structurally distinct strain of self-renewing α-syn fibrils in mice capable of initiating hallmark patterns of synucleinopathy similar to that induced by the wild-type form of α-syn commonly observed in PD (Kim et al., 2016). The modulation of α-syn amyloidogenesis caused by interaction with LPS has been characterized as a heteromolecular interaction resulting in the formation of intermediate nucleating species which then mature into divergent fibrillar forms of α-syn, affecting both cellular internalization and related cytotoxicity (Bhattacharyya et al., 2019).

Several studies have explored the potential connection between α-syn-related pathology and GI symptoms of PD. While α-syn is found copiously in the brain, it is also found in the ENS and is produced by enteric neurons to mediate neurotransmitter release and uptake (Grathwohl et al., 2013). In individuals with PD, pathological α-syn aggregates have been found in GI tissue biopsies (Sánchez-Ferro et al., 2015), lending support to the theory that PD pathology could be initiated in the ENS. Importantly, α-syn has also been found in the salivary glands, esophagus, and stomach (Fayyad et al., 2019), potentially corresponding to common non-motor symptoms such as hypersalivation, dysphagia, delayed gastric emptying, and gastroparesis. In a study involving a transgenic mouse model of PD, an amyloid precursor protein inhibitor, Posiphen, was injected into the mice to inhibit the misfolding of α-syn (Kuo et al., 2019). Results of this study demonstrated normalized distal colon motility following treatment, an important factor associated with maintenance of a balanced microbiome – presenting a potential novel early intervention for PD, given the frequent early onset of GI symptoms related to gut motility such as constipation, which warrants further investigation. However, a recent study reported the sensitivity associated with the presence of α-syn in colon tissue biopsies of PD patients to be approximately 14% (8/57) (Chahine et al., 2020). A meta-analysis of 21 studies estimated the sensitivity and specificity of α-syn in the colon tissue to be approximately 57 and 82%, with an approximate pooled odds ratio of 10 compared to non-PD controls (Bu et al., 2019). Though presence of α-syn in the gut likely contributes to development and progression, it is neither necessary nor sufficient for onset of PD pathology and is not an adequate predictor of future disease.

Notably, Braak et al. (2003) and Hawkes et al. (2007) proposed potential involvement of the GI tract in the development and progression of PD. The authors hypothesized that an unknown pathogen may enter the body through the nasal cavity, and such a neurotropic pathogen could potentially induce conformational changes in normal α-syn molecules, thereby resulting in their aggregation. The pathogen could then enter via the gastric mucosa and subsequently the central nervous system (CNS) via retrograde transport. Braak and colleagues hypothesized that PD α-synucleinopathy begins in the periphery, gains access to the CNS via retrograde transport along vulnerable neuronal projections within the ENS, and ascends caudo-rostrally from the lower brainstem in various distinct stages (Braak et al., 2003; Hawkes et al., 2007; Visanji et al., 2013). This hypothesis is unique in that it proposes a potential outside source (e.g., pathogen) as the catalyst for the onset of PD pathology as opposed to an inherent or internally driven mechanism. However, this hypothesis and its related staging system has been historically criticized for its lack of applicability to patients outside of a particular subset of those with the sporadic form of PD. Evidence supports some aspects of this hypothesis such as the presence of α-syn aggregates in the vagus nerve and their ability to spread from the ENS to the CNS (Musgrove et al., 2019; Figure 1D) as well as the related prevention of this propagation following vagotomy in animal models of PD (Kim et al., 2019). Further, novel experimental models of PD have been developed through direct initial inoculation of α-syn in the GI tract that subsequently induces synucleinopathy pathology in the CNS (Uemura et al., 2018; Kim et al., 2019; Challis et al., 2020). Nevertheless, recent results from a whole-body autopsy series of PD, incidental Lewy body disease, and otherwise healthy control participants (*N* = 187) failed to demonstrate a single case in which there was Lewy pathology in the gut without Lewy pathology also present in the brain (Beach et al., 2021). Therefore, further research is warranted to establish whether Lewy body pathology may originate in the gut independently from that of the brain, and whether this pathology in the gut is associated with clinically significant differences in disease-state or health outcomes related to PD compared to those without gut involvement to determine its potential utility as a novel intervention method.

## Microbiota Composition and Metabolites Associated With Parkinson’s Disease and Symptom Severity

The microbiome is the collection of all individual microbes (bacteria, fungi, viruses, and single-celled organisms), forming complex microbiota, and their genomes within a particular anatomic site (Ursell et al., 2012). It is dynamic across the lifespan and is influenced by a multitude of factors including, age (Badal et al., 2020), lifestyle factors including diet (Frame et al., 2020) and exercise patterns (Bycura et al., 2021), and medication use (Vich Vila et al., 2020). The GI tract in mammals contains a complex and symbiotic ecosystem with diverse interactions between the GI epithelium, immune cells, and commensal microbes. The gut microbiota are thought to be key mediators in the bi-directional communication between the gut and the brain along the gut–brain axis that influence neurodevelopment, brain function, and behavior (Yang et al., 2019). An undesirable shift in microbiota composition, termed dysbiosis, has been associated with both GI and metabolic diseases such as inflammatory bowel disease (Greenblum et al., 2012), obesity (Turnbaugh et al., 2006), and diabetes (Qin et al., 2012). Gut dysbiosis has also been observed in various neurological and psychiatric conditions including autism spectrum disorder (Golubeva et al., 2017), major depression (Foster and Neufeld, 2013), PD (Scheperjans et al., 2015a), and Alzheimer’s disease (Vogt et al., 2017). Certain gut microbiota composition dynamics have been associated with specific diseases or conditions across a broad population (e.g., increased relative abundance of the *Akkermansia* genera over time among individuals with PD across multiple geographic locations (Finland, Germany, Japan, Russia, and United States) (Nishiwaki et al., 2020b). However, defining a generalizable “normal” or “healthy” gut microbiome across the lifespan remains an important challenge due to both natural interindividual differences and intraindividual changes in microbiota composition over time.

A study analyzing 190 colonic tissue samples from participants with and without GI-related conditions (e.g., Crohn’s disease, ulcerative colitis, and controls being treated for cancer) estimated the average gut microbiome to be comprised of up to 35,000 species of bacterial and archaeal species (Frank et al., 2007); approximately 90% appearing to belong to the phyla Bacteroidetes and Firmicutes (Rinninella et al., 2019). Commensal gut microbiota play an important role in maintaining homeostasis and preventing certain pathologies, partly through production of vital micronutrients and fermentation of otherwise non-digestible polysaccharides into SCFAs (e.g., acetate, propionate, and butyrate) (Wells et al., 2017). SCFAs serve as a source of energy for host tissue, function as signaling molecules to regulate the expression of tight junction proteins of the intestinal epithelial barrier and play a role in the modulation of host immunity (Mathewson et al., 2016). Additional work has demonstrated that butyrate plays a role in regulating immune response via expansion of Treg cell populations (Arpaia et al., 2013; Furusawa et al., 2013), which is suggestive of a role for SCFA in ameliorating pro-inflammatory responses of immune cells to antigenic stimuli. Certain microbes, such as *Faecalibacterium prausnitzii*, help maintain homeostasis through production of SCFAs (butyrate) and modulation of downstream inflammatory pathways through potential induction of a tolerogenic cytokine profile (e.g., low secretion of proinflammatory and elevated secretion of anti-inflammatory cytokines) (Rossi et al., 2015; Lenoir et al., 2020). A recent microbiome-wide association study using two large datasets (*N* = 333; *N* = 507) observed an elevation of opportunistic pathogens, while potentially beneficial bacteria able to metabolize carbohydrates (precursors for SCFAs) among individuals with PD, were depleted in this same population (Wallen et al., 2020). Further, compared to conventionally colonized controls, GF mice (mice raised under sterile conditions or depleted of their intestinal microbiota through administration of oral broad-spectrum antibiotics) exhibit substantial physiological alterations, such as increased BBB permeability, that are relevant to neurodegenerative disorders such as PD. Depletion of the gut microbiota through administration of antibiotics was associated with statistically significant improvement in various motor symptoms (duration and severity of dyskinesia, duration of medication “off” state, functional impact of motor functions, and complexity of motor fluctuations) among 14 individuals with PD (Baizabal-Carvallo et al., 2021).

Non-motor symptoms, including GI disturbances such as constipation, have often been found to considerably precede the onset of hallmark neurological features of PD (Klingelhoefer and Reichmann, 2015). Further, over 80% of PD patients report some level of GI dysfunction throughout the course of their disease (Pfeiffer, 2011). Further, according to the Honolulu Heart Program study, males between 51 and 75 years of age who passed less than one bowel movement per day were 2.7 times more likely to develop PD compared to males of the same age range who passed at least one bowel movement per day (Abbott et al., 2001). Although research on the relationship between the GI system and neurodegenerative diseases is still in its early stages, several promising hypotheses have been developed. The gut microbiome is thought to play a role, in at least a subset of PD patients, in both the initiation and progression of PD pathology and symptomology (Gorecki et al., 2019; Yang et al., 2019; Liu et al., 2020). Studies have shown a correlation between the increased occurrence of neurodegenerative diseases and conditions known to be associated with disrupted gut microbiota diversity such as insomnia, REM sleep behavior disorder (RBD), and constipation, with approximately 40–65% of RBD patients developing a neurodegenerative disorder within 10 years of RBD diagnosis (Postuma et al., 2009; Heinzel et al., 2021). Moreover, in a study of 172 RBD patients, 94% of those with associated comorbid neurodegenerative disease had a form of synucleinopathy (Boeve et al., 2013). Gut microbiota may modify the association between RBD and PD given the evidence from a recent study where there was an increase in the relative abundance of genus *Akkermansia*, a genus of mucin-metabolizing bacteria (Earley et al., 2019) that are commonly elevated among individuals with PD (Romano et al., 2021), in individuals with RBD (Heintz-Buschart et al., 2018; Nishiwaki et al., 2020a).

Colonic motility follows a circadian rhythm similar to that of the suprachiasmatic nucleus in the brain and can be become desynchronized due to changes in a variety of external stimuli including light exposure, food intake patterns, and exercise (Duboc et al., 2020). Sleep disturbances are also known to be associated with potentially pathological mechanisms (e.g., proinflammatory pathway expression, stress on the endoplasmic reticulum, abnormal proteostasis, impaired glymphatic clearance, nocturnal cerebral hypoxia, and altered modulation of specific neural circuits) that may increase the risk for PD (Bohnen and Hu, 2019). As pathological α-syn has been observed to propagate along the brain’s inherent neural networks (e.g., network-spread hypothesis), sleep disturbances that affect related neural networks may influence either the development or transmission of α-syn aggregates in the brain (Yau et al., 2018). Therefore, sleep-related disorders may be indirectly associated with risk of PD and may result from pathological mechanisms associated with sleep disturbance, with maladaptive shifts in the microbiota being both a possible product of and a risk factor for this potential pathway (e.g., positive feedback loop).

Various studies over the past few decades have observed distinct differences in microbiota composition between individuals with and without PD, as well as between individuals with different PD phenotypes of symptomology (Heintz-Buschart et al., 2018; Heinzel et al., 2021). A recent study observed differences in the microbiota (elevated *Akkermansia*, *Eggerthella*, and *Synergistetes*; depleted *Prevotella*) between A53T transgenic monkeys with early-stage PD and control monkeys (Yan et al., 2021a). These results extend upon commonly observed microbiota differences between human PD patients and healthy controls to an earlier timepoint of disease progression. Generally, studies have found that those with PD have a higher relative abundance of bacteria from the genera *Akkermansia*, *Lactobacillus*, and *Bifidobacterium*, and lower relative abundances of *Prevotella*, *Faecalibacterium*, *Bacteroidetes*, and *Blautia* genera (Keshavarzian et al., 2015; Scheperjans et al., 2015a; Li et al., 2017; Barichella et al., 2019). These differences in relative abundance have been linked to outcomes associated with the disease and could be considered dysbiosis (a deleterious microbiota composition characterized by the loss or gain of bacteria that promote health or disease, respectively (Wilkins et al., 2019) in this population. Specifically, *Lactobacillus*, *Enterococcus*, *Escherichia*, and *Proteus* genera have been positively associated, and *Blautia*, *Faecalibacterium*, and *Ruminococcus* have been negatively associated with the Unified Parkinson’s Disease Rating Scale (UPDRS – a scale that measures intellectual function, mood, behavior, ability to perform activities of daily living, and motor functionality and complications), respectively (Li et al., 2017; Barichella et al., 2019). Members of the *Lachnospiraceae* family have been found to be negatively associated with postural instability and gait disturbances (Barichella et al., 2019) and those of *Enterobacteriaceae* have been positively linked to general symptom severity (Scheperjans et al., 2015a). Further, inflammatory biomarkers and SCFAs in the stool are both inversely associated with microbial alpha diversity in the gut, with some bacterial taxa being directly correlated with SCFA levels (↓SCFAs: *Akkermansia*, *Escherichia/Shigella*, *Flavonifractor*, *Intestinimonas*, *Phascolarctobacterium*, *Sporobacter*; ↑SCFAs: *Butyricicoccus*, *Clostridium* sensu stricto, *Roseburia*). However, it should be noted that these relationships are correlational and are likely influenced by a multitude of both disease-specific and non-specific internal and external factors such as medication type and dose, changes in physical activity and diet, and changes in gut transit time related to PD pathology. Additionally, it is not yet known whether these relationships are simply a product of physiological alterations, either due to changes in external factors associated with the disease (e.g., behavioral or diet changes, use of medications, etc.) or pathological disease progression, or whether they may be indicative of the microbiota being a true contributing factor of PD. Given the complex bidirectional relationship between the microbiome and host health in addition to the heterogeneity in clinical phenotypes of PD (Markello et al., 2021), is possible that microbiota dynamics are both a product PD pathology as well as potential drivers for the onset and progression of the disease.

The influence of gut microbiota on its host is determined both by composition and abundance, which are influenced by a variety of internal and external factors, including host genetics, age, dietary and lifestyle habits, and antibiotic and other medication use (Hasan and Yang, 2019). Evidence suggests that gut microbiota indicative of good health have the ability to positively regulate neuroimmune response in the CNS, and that bacterial dysbiosis (e.g., maladaptive shifts in the microbiota composition and/or abundance) could promote a neuroinflammatory response, increasing the risk for the development of neurodegenerative diseases (Sampson and Mazmanian, 2015). Dysbiosis may lead to compromised gut barrier integrity, subsequent microbial translocation (Figure 1C), and disruption of the neuroendocrine system through the production of LPS and upregulation of proinflammatory cytokines (Mulak and Bonaz, 2015). Certain bacteria can specifically signal enteric dopaminergic neurons through neuronal uptake of secreted bacterial amyloids and neurotransmitters, thereby influencing intestinal mobility and secretion through indirect action on vagal afferent fibers (Bonaz et al., 2018). The presence of bacterial overgrowth and dysbiosis may be detected by increased urinary indoxyl sulfate and possibly low abundance of fecal Prevotellaceae (Cassani et al., 2015; Scheperjans et al., 2015a), a bacterial family often found to be depleted in PD and inversely correlated with motor score severity on part 3 of the UPDRS (Gerhardt and Mohajeri, 2018). However, the metabolic capabilities of *Prevotellaceae*, and which specific members of this bacterial family may play a role in PD, are not well understood.

## Microbiota, Barrier Permeability, and Parkinson’s Disease Pathology

The relationship between certain gut microbiota and increased intestinal barrier permeability is noteworthy as compromised barrier integrity has recently been observed in PD patients and could potentially contribute to the misfolding of α-syn proteins (Forsyth et al., 2011; Clairembault et al., 2015). One study found increased levels of fecal calprotectin zonulin and alpha-1-antitrypsin in PD patients compared to age-matched controls, indicating increased intestinal inflammation and disrupted barrier function in this population (Schwiertz et al., 2018). In another study involving sigmoid biopsies of patients with PD, investigators found decreased expression of tight junction proteins ZO-1 and occludin, as well as irregular distribution of these proteins in the tissue biopsies, in PD patients compared to non-PD controls (Clairembault et al., 2015). To our knowledge, only four studies have investigated intestinal permeability in PD compared to matched controls, three of which used a non-invasive orally administered sugar probe as opposed to direct colonic tissue biopsies. Notably, one of these studies found intestinal permeability in PD patients to be significantly correlated with intestinal expression of α-syn, presence of *Escherichia coli* in the gut, and increased levels of LPS-binding proteins in serum (Forsyth et al., 2011). Gut microbiota not only mediate intestinal inflammation and permeability within the GI tract, but may also influence expression of α-syn within the brain due to changes in permeability of the BBB (Braniste et al., 2014; Fröhlich et al., 2016).

Despite all the evidence pointing to the potential link between gut bacteria, inflammation, and PD, the underlying molecular mechanisms remain elusive. PINK1 (kinase) mutations are implicated in PD etiology and PINK1 likely plays a role in immune function and is instrumental in mitochondrial dynamics (Gonçalves and Morais, 2021). Matheoud et al. (2016) reported that PINK1 can suppress antigen presentation derived from degraded mitochondria induced by LPS exposure. Recently, Matheoud et al. (2019) also proposed that in the absence of PINK1, mitochondrial antigen presentation resulting from intestinal infection and insult can lead to dopamine neuron dysfunction (Herrick and Tansey, 2019; Matheoud et al., 2019). These results are consistent with findings where motor deficits in PD patients worsen during peripheral infections (Umemura et al., 2014). Taken together, these results support the notion that PINK1 is a repressor of the immune system and that intestinal infection may be a trigger for PD, which supports the role of the gut–brain axis in this disease (Houser and Tansey, 2017).

## Potential Mechanisms Underlying Gut–Brain Axis Communication in Parkinson’s Disease

The bidirectional pathway between the gut and the brain has been of interest for several decades and a variety of methods have been utilized to investigate this axis including infection studies, germ-free (GF) animal models, and intervention studies (e.g., prebiotics, probiotics, antibiotics) (Bravo et al., 2012). It is thought that commensal bacteria can both indirectly and directly influence PD pathology through the circulatory and nervous systems, both enteric and central. The neural communication for the GI tract involves a multi-tier network beginning with the myenteric submucosal plexus and enteric glial cells. Most notably, catecholaminergic neurons are most tightly juxtaposed to the lumen of the gut (Chesné et al., 2019). The vagal nerve directly innervates the myenteric plexus, and these neurons lead to prevertebral ganglia within the spinal cord and finally to higher brain centers. Terminals of the vagal afferent neurons, which are positioned within the gut mucosa, directly convey information to the brain and have been shown to be responsive to LPS (de La Serre et al., 2015), an endotoxin produced by Gram-negative bacteria. High levels of LPS have been shown to activate vagal afferent neurons, resulting in hypophagia (reduction in food intake and eating behavior) and weight loss – common non-motor symptoms of PD (Gakis et al., 2009). In PD, certain microbiota compositions (e.g., dysbiosis) have been found to stimulate the production of inflammatory cytokines and LPS, leading to intestinal epithelial damage and compromised barrier integrity (van IJzendoorn and Derkinderen, 2019). Increased intestinal permeability results in microbial translocation and the introduction of bacterial-derived toxins and host-derived inflammatory cytokines (TNF, IL-6, IL-1) into the blood stream (Figure 1C), providing an avenue for direct interaction with the nervous system mediated by compromised integrity of the BBB (Obrenovich, 2018). These cytokines have been found to be significantly elevated in the serum of individuals with PD compared to healthy controls and may correlate with symptom severity and progression of disease (Brodacki et al., 2008; Rentzos et al., 2009; Eidson et al., 2017; Houser and Tansey, 2017; Kim et al., 2018; Rathnayake et al., 2019).

Studies involving the maturation of GF animals have provided evidence supporting the theory that the gut microbiota are essential for the appropriate development of both the ENS and CNS (Stilling et al., 2014). Further, the complete absence of bacterial colonization in the gut of these GF animals has been associated with distinct alterations in the functionality of the CNS and ENS including delayed gastric emptying, increased intestinal transit time, reduced migrating myoelectric complex periods (normal cyclic motility patterns which occur in the stomach and small intestines during periods of fasting), enlarged cecum, altered gene expression related to regulation of neurotransmitters, muscular contractile proteins, and brain-derived neurotrophic factor (BDNF) (Carabotti et al., 2015). Animals raised in GF conditions have also demonstrated increased BBB permeability through decreased expression of tight junction proteins, potentially due to a lack of bacterial-derived SCFAs, capable of improvement following colonization with commensal microbiota (Ma et al., 2019). Taken together, these studies suggest the inherent presence of a bidirectional pathway between the gut and the brain, mediated by the microbiota, is vital for proper nervous system maturation and function.

## The Vagus Nerve as a Connection Between the Gut and Brain

The vagus nerve is a critical component in the connection between the gut and the brain, through either direct interaction with the microbiota or indirect interaction mediated through microbiota communication with enteroendocrine cells (Figure 1B) and subsequent signaling to afferent neurons via hormone production (e.g., cholecystokinin, 5-hydroxytryptamine, peptide YY, etc.) (Bellono et al., 2017). Several retrospective studies have explored the possible link between history of vagotomy (removal of a portion of the vagus nerve thereby severing the neural connection between the gut and the brain) and the risk for developing PD. One large retrospective cohort study involving over 14,000 previously vagotomized patients observed a decreased risk, though not reaching significance, for developing PD in patients who had a specific type of vagotomy, known as a truncal vagotomy (division of the anterior and posterior vagal trunks) (Svensson et al., 2015; Liu et al., 2017). However, another similar study including 9,430 vagotomized patients and matched controls over a cumulative 7.3 million person-year follow-up period produced conflicting results revealing no association between vagotomy and risk for PD (Liu et al., 2017). Further, other recent studies have suggested systemic brain-to-gut communication that bypasses the vagus nerve. Arotcarena et al. (2020) found evidence of either caudo-rostral or rostro-caudal spread of pathological α-syn in a non-human primate model of PD with no pathological lesions observed in the vagal nerve. An additional study using a rat model of PD found evidence of pathological changes originating from nigral overexpression of α-syn (e.g., significant neuronal loss in ileal submucosal plexus, increased glial expression in myenteric plexus, alterations in gut microbiome composition and bile acid metabolism), further supporting the brain-to-gut propagation that may be entirely independent of the vagus nerve (O’Donovan et al., 2020). Additional research is necessary to definitively characterize the potential modes of pathological α-syn propagation in PD to inform potential future screening or therapeutic options.

## Gut Microbiota and Efficacy of Immunotherapies and Medications for Parkinson’s Disease

While immunotherapy is now frequently used for the treatment of cancer, there has been recent interest in the potential utility of this approach for PD, and other neurodegenerative diseases known to be mediated by host immune function. Such immunotherapy targets elevation of regulatory T cell counts and upregulation of regulatory T cell function, which are both commonly significantly diminished among individuals with PD (Williams-Gray et al., 2018; Yan et al., 2021b). Sulzer et al. (2017) showed that in PD, there is T-cell recognition of defined epitopes, which are derived from α-syn, presented by certain Major Histocompatibility Complex (MHC) alleles. This is capable of driving both helper and cytotoxic T-cell responses in PD patients. Further, T cells that are specifically reactive to α-syn have been associated with preclinical and early stages of PD (Lindestam Arlehamn et al., 2020). The metabolites of certain gut bacteria, particularly butyrate, have recently been observed to improve efficacy of certain cancer therapeutics through upregulation of CD8+ T cells within the microenvironment, both *in vitro* and *in vivo* (He et al., 2021). Additionally, a recent study observed an inverse association between microbial diversity (artificially depleted with an antibiotic cocktail) and intratumor specific immune responses derived from injection with a neoantigen cancer vaccine (Lione et al., 2021). However, the mechanisms underlying the relationship between gut microbiota dynamics and adaptive immune response in animal models of disease are yet to be determined. The results of these recent studies support the hypothesis that microbiota and associated metabolites modify therapeutic efficacy and health outcomes through modulation of the host immune system.

Considering the connection between microbiota and host immune function, we conducted a study to evaluate gut microbiota dynamics associated with a novel adoptive cellular therapy intervention using α-syn-specific T cells and observed distinct clustering of the microbiota between animals in the treatment and control (injection with a novel adoptive cellular therapy or saline placebo) groups post-baseline, respectively (Klann et al., 2020). Further, specific bacterial taxa found to be differentially abundant among the animals in the treatment arm compared to the control arm, such as members of the *Odoribacter* genus, are known to produce butyrate (SCFA known to play a role in immune modulation and gut epithelial barrier integrity). These results suggest that the gut microbiota are associated with immunotherapy in the context of PD. However, whether observed microbiota dynamics influence outcomes related to the immunotherapy or are conversely a product of the therapy and/or progression of PD pathology is not clear.

Gut microbiota have also been found to influence the ability of the host to uptake certain orally delivered pharmaceuticals due to their functional capabilities and metabolic processes. More specifically, recent research suggests that gut microbiota composition contributes to the variability of L-dopa efficacy among PD patients as certain bacterial species (*Enterococcus faecalis* and *Eggerthella lenta A2*) are known to metabolize this compound prior to crossing the BBB, rendering it inactive and ineffective (Rekdal et al., 2019). Further, common PD drugs, such as levodopa and LD-carbidopa intestinal gel, have been found to induce changes in both the composition and functional metabolic capacity of the gut microbiota (Melis et al., 2021). In a study including 107 PD patients categorized into groups by pharmaceutical regimen (levodopa: *N* = 46, LD-carbidopa intestinal gel: *N* = 38, antiparkinsonian medication naïve: *N* = 23), differences in microbiota composition were observed both between medication groups and the medication naïve group (Melis et al., 2021). Among those in the LD-carbidopa intestinal gel group, the relative abundance of *Enterobacteriaceae*, *Escherichia*, and *Serratia* was increased compared to the levodopa group, and *Proteobacteria* and *Enterobacteriaceae* were found to be in higher abundance and *Firmicutes*, *Lachnospiraceae*, and *Blautia* were found to be reduced in relative abundance compared to the naïve group. *Blautia* and *Lachnospiraceae* were also observed to be reduced among the levodopa group compared to the naïve group. Further, both medication groups were associated with a metabolic profile linked to intestinal inflammation. This evidence suggests a complex relationship between the gut microbiota and PD treatment outcomes in which the overall composition is influenced by common PD pharmaceuticals while specific taxa are also directly involved in the metabolism and uptake of these same pharmaceuticals. The microbes known to metabolize PD pharmaceuticals and decrease their efficacy may represent a promising target for methods aiming to improve patient outcomes related to certain medications. Further, consistent alterations in the gut microbiota composition in response to certain PD medications may inform the development of a potential probiotic supplement to counteract any maladaptive changes linked to worsened symptom severity or disease progression. A recent study including 197 PD cases and 130 controls revealed distinct and statistically significant microbial signatures associated with catechol-*O*-methyltransferase-inhibitors, anticholinergics, and carbidopa/levodopa medications (Hill-Burns et al., 2017). Therefore, the influence of certain medications on the commensal microbiota is also an important factor and potential confounder of the relationship between the microbiome and PD. A homogeneous medication regimen (e.g., medication type, dosage, length of time using medication, etc.) among PD patients would be ideal for valid interindividual comparisons for microbiome studies in this population. Additionally, bacteria previously determined to be associate with PD medication should be considered when characterizing changes in the microbiota related to PD pathology.

## Microbiota-Targeted Interventions for Parkinson’s Disease

Supplementation with pre- and probiotics during adolescence could improve resiliency toward the development neurodegenerative disorders through modulation of various biomolecules known to reduce inflammation and promote neurogenesis (ferulic acid), suppression TNF production and decrease TLR signaling (histamines), reduction of reactive oxygen species accumulation, and improvement of synaptic plasticity (ghrelin) (Yahfoufi et al., 2020). In the context of PD, probiotics are thought to potentially improve symptoms through altering composition of the gut microbiota to reverse dysbiosis and disrupt pathways related to inflammation (Castelli et al., 2020) and microbial translocation or “leaky gut” (Figure 1E; Ghyselinck et al., 2021). Recently, it has been found that, in a *C. elegans* model of synucleinopathy, supplementation with the *Bacillus subtilis* probiotic strain PXN21 actively cleared pre-existing α-syn aggregates as well as conferred protection against the formation of additional aggregations; however, the researchers note that this protection was likely partially mediated by model-specific DAF-16 gene expression (Goya et al., 2020). While there are limited human clinical trials investigating probiotic supplementation for the treatment or management of PD, a few pilot studies have been conducted for the treatment of specific PD-related symptoms, such as constipation, in this population. Recent clinical trials suggest that probiotics can be useful in ameliorating GI issues such as constipation in patients with PD (Cassani et al., 2011; Barichella et al., 2016; Tan et al., 2021), and this is a growing area of research. One recent randomized, double-blind placebo-controlled trial of 120 participants with PD and Rome-III-confirmed constipation was conducted to assess the potential use of probiotics to improve constipation (Barichella et al., 2016). Participants consumed a fermented milk beverage, containing prebiotic fiber fructooligosaccharides (FOS) and 250 × 109 colony forming units (CFUs) of a probiotic combination (*Streptococcus salivarius* subsp. *thermophilus*, *Enterococcus faecium*, *Lactobacillus rhamnosus GG*, *L. acidophilus*, *L. plantarum*, *L. paracasei*, *L. delbrueckii* subsp. *bulgaricus*, and *B. breve and B. animalis* subsp. *lactis*), or a nutritionally similar placebo not containing the pre- and probiotic mixture, once daily for 4 weeks. The authors found that the fermented milk, fortified with pre- and probiotics, effectively increased the number of complete bowel movements, total number of bowel movements, and improved stool consistency (as measured by the Bristol Stool Scale) among individuals with PD. Participants in the treatment arm of this study also reported a decreased need for the use of laxatives, medications frequently used to decrease the severity of rigidity and increase bowel movement frequency (Augustin et al., 2016). Another randomized double-blind placebo-controlled trial investigated the impact of probiotic supplementation on movement and metabolic parameters in PD (Tamtaji et al., 2019). In this study, participants were randomized to receive either a probiotic combination (*L. acidophilus*, *B. bifidum*, *L. reuteri*, and *L. fermentum*, each 2 × 109 CFUs) or a placebo once daily for 12 weeks. Individuals in the probiotic combination arm experienced an average reduction of approximately five points on the UPDRS compared to an average increase of approximately four points experienced by the placebo arm. However, results did not include changes in subcomponents of the MDS-UPDRS; therefore, a concise conclusion related to the decrease in motor versus non-motor symptoms cannot be determined. While the direction of the association is promising, considering the UPDRS range of 0–195 points, a five-point reduction may not equate to a clinically or biologically relevant change. However, those in the probiotic arm also demonstrated significant reductions in free radicals and oxidative stress [known risk factors for α-syn and other protein misfolding (Scudamore and Ciossek, 2018)], C-reactive protein (proinflammatory biomarker), insulin concentrations, and insulin resistance compared to those in the placebo arm. Another potential mechanism for regulating gut microbiota is through the hypothalamic–pituitary–adrenal (HPA) axis, an important pathway in the study of PD due to its involvement in immune responses to stress and inflammation. A study using GF mice found that inoculation with *Bifidobacterium infantis* effectively counteracted the stress-induced upregulation of the HPA axis (implemented through exposure to acute restraint stress), potentially through undetermined neural- or cytokine-mediated pathways (Miraglia and Colla, 2019). Such regulatory pathways highlight the potential influence of the microbiome on the modulation of stress and production of inflammatory hormones, such as cortisol. Therefore, probiotics may be a promising option to improve common symptoms of PD while also reducing levels of systemic inflammation through modulation of inflammatory pathways.

Individuals with PD also commonly experience comorbid affective disorders such as anxiety, depression, and apathy (Aminian and Strafella, 2013). While no studies specific to PD have investigated treatment of these concurrent conditions by targeting the gut–brain axis, others have been conducted for different neurological disorders. In one such study, the probiotic *Bifidobacterium breve* strain A1 was administered to patients with schizophrenia and resulted in a 25% average decrease in symptoms related to anxiety and depression (Okubo et al., 2019). Similar results were found in a study evaluating the impact of prebiotics in children with Autism Spectrum Disorder (Grimaldi et al., 2018). It is hypothesized that similar treatment methods may be beneficial in reducing psychological manifestations (e.g., depression, anxiety, apathy, psychosis, impulsive or compulsive behaviors, etc.) associated with PD. While the underlying mechanisms of this potential association are not well understood, several pathways have been proposed. Certain microbial metabolites directly interact with enteroendocrine cells (Figure 1B) and trigger the release of various endocrinologically active compounds including cholecystokinin and glucagon-like peptide 1, which may initiate changes in host physiology and behavior (Noonan et al., 2020). For example, glucagon-like peptide 1 has also been found to have a preventive effect on the progression of Alzheimer’s disease pathology through suppression of TNF levels and protection against synaptic dysfunction related to LPS in a murine model (Kim et al., 2020). Similarly, cholecystokinin is thought to activate neurons in the hindbrain and intestinal myenteric plexus and is necessary component for long-chain fatty acids [compounds associated with gut motility in a rat model (Zhao et al., 2018)] to interact with vagal afferent nerves (Breit et al., 2018).

Other intervention methods targeting the microbiome–gut–brain axis to improve symptoms related to PD include antibiotics, fecal microbiota transplantation, and dietary interventions. While the use of antibiotics as a potential intervention is undoubtedly associated with adverse effects (e.g., propagation of antibiotic resistance, induction of further dysbiosis due to a potential decrease in microbial diversity), there has been recent interest concerning the effects of certain antibiotics, such as anti-inflammatory or antioxidant properties associated with Doxycycline (Sultan et al., 2013; Santa-Cecília et al., 2019), in neurodegenerative disorders to potentially restore balance in microbiome composition (Gazerani, 2019). Further, Minocycline has been linked to neuroprotective effects in an MPTP mouse model of PD through its ability to cross the BBB and prevent the loss of nigrostriatal dopaminergic neurons (Du et al., 2001). It has also been suggested to influence the gut–brain axis through modulation of toll-like receptor 4 (TLR4), an important transmembrane protein present in the intestinal epithelium known to be activated by LPS derived from Gram-negative bacteria (Velloso et al., 2015). Other studies have suggested that increased abundance of particular microbiota, such as *Escherichia coli* (Forsyth et al., 2011) and *Ralstonia* species (Keshavarzian et al., 2015), is associated with ENS inflammation and increased levels of endotoxins and proinflammatory cytokines (e.g., TNF, IFN-gamma, interleukins, and activation of enteric glial cells) (Yang et al., 2019), which may be diminished through the use of either antibiotics or bacteriophages.

Fecal microbiota transplantation, a technique used to transfer feces from a healthy donor to the GI tract of a recipient, has been used to restore the commensal gut microbiota and has proven effective for treating recurrent infections with *Clostridium difficile*. This technique has also been evaluated for use in the treatment of other conditions unrelated to the GI tract including multiple sclerosis (Makkawi et al., 2018) autism (Kang et al., 2019), and amyotrophic lateral sclerosis (ALS) (Mandrioli et al., 2019), and has recently been found to alleviate symptoms associated with Alzheimer’s disease in APP/PS1 transgenic mice (Sun et al., 2019). Lastly, dietary and lifestyle interventions have been of interest as a potential complementary intervention for improvement of PD symptoms. Recent evidence suggests that various lifestyle factors, including caffeine consumption and following a Western diet (e.g., high caloric intake, high levels of saturated and omega-6 fatty acids, excessive salt and sugar intake, etc.), may alter the gut microbiota and therefore mediate both the risk of developing PD and the clinical progression of previously diagnosed PD (Scheperjans et al., 2015b; Jackson et al., 2019).

Another potential method of interest in preventing or delaying the onset of PD is the utilization of molecular mimicry, a concept explaining the structural similarities in secreted proteins between microorganisms and their respective hosts. Some researchers hypothesize that α-syn may be recognized as a microbe-associated molecular pattern (MAMP) mimicking bacterial amyloids as α-syn has been observed to bind to toll-like receptor 2 (TLR2; a pattern recognition receptor important for pathogen recognition) on microglia (Kim et al., 2013). Interestingly, a recent study in Thy1-αSyn mice found that gut bacterial amyloid proteins, chiefly expression by curli proteins derived from *Escherichia coli*, promote the aggregation of α-syn in both the gut and the brain, resulting in behavioral deficits, intestinal dysfunction, and motor impairments (Sampson et al., 2020). In another study, a mixture of SCFAs was injected into α-syn transgenic mice leading to neuroinflammation, damage to dopaminergic neurons, and motor dysfunction (Miraglia and Colla, 2019). These mice were then treated with minocycline, which targets TNF activation, therefore reducing aggregation of misfolded α-syn and improving motor function. Although anti-TNF drugs have been used for several decades as a treatment for inflammatory diseases such as inflammatory bowel syndrome, they have just recently been applied to neurodegenerative disorders per exploration of their potential role within the gut–brain axis (Parameswaran and Patial, 2010). Another such study among individuals with inflammatory bowel syndrome (144,018 individuals with IBD and 720,090 matched controls) reported a 78% reduction in the incidence of PD among the population when anti-TNF therapy was previously utilized to treat symptoms related to the inflammatory bowel syndrome compared to those who were never exposed to anti-TNF therapy (Peter et al., 2018). Prevention or attenuation of inflammation in both the gut and the brain, through potential pathways, such as those outlined above, may confer protection against the development or progression of neurodegenerative disorders such as PD. Nevertheless, the currently FDA-approved anti-TNF inhibitors are also immunosuppressive which warrants caution when considering this approach in elderly populations. Results from several recent human and murine studies strongly suggest that gut microbiota may be modified, through the supplementation of certain pre- and probiotic combinations, to improve various clinical endpoints associated with PD [e.g., improvement in GI-related symptoms and motor coordination, decreased levels of inflammatory biomarkers and microglia activation, decreased MDS-UPDRS score, etc. (Tables 1, 2)].

**TABLE 1:** Summary of recent pre- and probiotic studies related to Parkinson’s disease in humans.

Study design	Sample size	Pre/probiotic	Frequency of use	Main findings	Author and year
Randomized controlled trial	40 PD patients in probiotic arm	65 mL fermented milk drink containing 6.5 × 109 CFU of *Lactobacillus casei* Shirota daily	Once per day for 5 weeks	↑ Number of days with normal stool consistency; ↓ number of days feeling bloated, abdominal pain, and incomplete colon emptying	Cassani et al., 2011
Double-blind, randomized, placebo-controlled trial	34 PD patients in probiotic arm; 38 PD patients in placebo arm	10 billion CFU of *Lactobacillus acidophilus*, *L. gasseri*, *L. reuteri*, *L. rhamnosus*, *Bifidobacterium bifidum*, *B. longum*, *Enterococcus faecalis*, *E. faecium*	Once per day for 4 weeks	↑ Number of spontaneous bowel movements, improvement of stool consistency and quality of life related to constipation	Tan et al., 2021
Double-blind, randomized, placebo-controlled trial	80 PD patients in pre/probiotic mixture arm; 40 PD patients in placebo arm	250 × 10^9^ CFU of *Streptococcus salivarius* subsp. *thermophilus*, *Enterococcus faecium*, *Lactobacillus rhamnosus GG*, *L. acidophilus*, *L. plantarum*, *L. paracasei*, *L. delbrueckii* subsp. *bulgaricus*, *Bifidobacterium* (*breve* and *animalis* subsp. *lactis*)	Once per day for 4 weeks	↑ Number of complete bowel movements per week	Barichella et al., 2016
Double-blind, randomized, placebo-controlled trial	30 PD patients in probiotic arm; 30 PD patients in placebo arm	2 × 10^9^ CFU/g each of *Lactobacillus acidophilus*, *Bifidobacterium bifidum*, *Lactobacillus reuteri*, and *Lactobacillus fermentum*	Once per day for 12 weeks	↓ MDS-UPDRS, C-reactive protein, insulin, and malondialdehyde levels; ↑ glutathione levels, insulin sensitivity	Tamtaji et al., 2019
Randomized placebo-controlled trial	40 total PD patients, all increased water (2 L/day) and fiber intake (20–25 g/day); 20 patients in trimebutine arm; 20 patients in probiotic arm	60 mg of *Lactobacillus acidophilus* and *Bifidobacterium infantis*	Trimebutine: 200 mg, 3 times per day for 3 months Probiotic mixture: 2 times per day for 3 months	Trimebutine: ↓ abdominal pain, bloating, constipation, incomplete defecation Probiotic mixture: ↓ abdominal pain and bloating	Georgescu et al., 2016
Double-blind, randomized, placebo-controlled trial	25 PD patients in probiotic arm; 25 PD patients in placebo arm	2 × 10^9^ CFU each of *Lactobacillus acidophilus*, *Bifidobacterium bifidum*, *L. reuteri*, and *Lactobacillus fermentum*	Once per day for 12 weeks	↓ Expression of IL-1, IL-8, TNF (inflammatory cytokines); ↑ expression of TGF-β and PPAR-γ (immunoregulation factors)	Borzabadi et al., 2018
Randomized placebo-controlled trial	22 PD patients in probiotic arm; 26 PD patients in placebo arm	Hexbio^®^: 30 × 10^9^ CFU (107 mg each) of *Lactobacillus acidophilus*, *Lactobacillus casei*, *Lactobacillus lactis*, *Bifidobacterium infantis*, and *Bifidobacterium longum;* 2% fructo-oligosaccharide prebiotic	2 times per day for 8 weeks	↑ Average bowel opening frequency; ↓ gut transit time	Ibrahim et al., 2020

**TABLE 2:** Summary of recent pre- and probiotic studies in animal models of Parkinson’s disease.

Study design	Sample size	Pre/probiotic	Frequency of use	Main findings	Author and year
Randomized controlled trial	15 male C57BL/6 mice with 6-OHDA lesions; 15 male C57BL/6 mice with no lesions	270 μl of SLAB51 (*Streptococcus thermophilus*, *Bifidobacterium longum*, *B. breve*, *B. infantis*, *Lactobacillus acidophilus*, *L*. *plantarum*, *L. paracasei*, *L. delbrueckii* subsp. *bulgaricus*, *L. brevis*)	Once per day for 2 weeks	Counteraction of 6-OHDA-induced effects; ↓ neuroinflammation	Castelli et al., 2020, p. 5
Randomized placebo-controlled trial	10 MitoPark PD mice in treatment group; 10 MitoPark PD mice in placebo group	10^10^ CFU of *Bifidobacterium bifidum*, *Bifidobacterium longum*, *Lactobacillus rhamnosus*, *Lactobacillus rhamnosus GG*, *Lactobacillus plantarum LP28*, *Lactococcus lactis* subsp. *lactis*	Once per day for 16 weeks	↑ Motor coordination, preservation of TH+ cells in SNpc; ↓ gait instability	Hsieh et al., 2020
Randomized placebo-controlled trial	10 male Wistar rats in probiotic and 6-OHDA arm; 10 male Wistar rats in 6-OHDA arm; 10 male Wistar rats in placebo/control arm;	2 × 10^9^ CFUs each of*Lactobacillus acidophilus*, *Bifidobacterium bifidum*, *Lactobacillus reuteri*, *Lactobacillus fermentum*	Once per day for 2 weeks	↑ Rotational behavior and cognitive function; ↓ lipid peroxidation and neuronal damage	Alipour Nosrani et al., 2021
Randomized placebo-controlled trial	8 groups (7 male C57BL/6 mice each): treatment and placebo groups for each individual probiotic strain, treatment and placebo groups for 3-strain mixture	8 ± 2 × 10^8^ CFU/mL each of *Lactobacillus plantarum CRL 2130*, *Streptococcus thermophilus CRL 807*, *Streptococcus thermophilus CRL 808*	Once per day for 3 weeks	↑ Motor skills, TH+ cell counts, IL-10 counts in serum/brain tissue; ↓ IL-6 and TNF counts in serum	Perez Visñuk et al., 2020
Randomized placebo-controlled trial	26 C57Bl6/J male mice in probiotic group; 26 C57Bl6/J male mice in dextran sodium sulfate group; 26 C57Bl6/J male mice in placebo group	VSL#3^®^: 5.4 × 10^9^ CFU of *Streptococcus thermophilus*, *Bifidobacterium breve*, *B. lactis*, *Lactobacillus acidophilus*, *L. plantarum*, *L. paracasei*, *L. helveticus*	Once per day for 4 weeks	↓ LPS- and paraquat-induced weight loss	Dwyer et al., 2021
Randomized placebo-controlled trial	6 groups (12 male C57BL/6 mice each): MPTP only, probiotic (10^7^ CFU) prior to MPTP, probiotic (10^7^ CFU) and MPTP simultaneously, probiotic (10^9^ CFU) prior to MPTP, probiotic (10^9^ CFU) and MPTP simultaneously, placebo group (no MPTP or probiotics)	10^7^ or 10^9^ CFUs of engineered probiotic (MG1363-pMG36e-GLP-1) that continually expresses GLP-1 (Chen et al., 2018)	Once per day for 1 week (pre- treatment groups); Once per day for 14 days (treatment for entire study period)	↓ MPTP-induced locomotor impairments, microglia and astrocyte activation, expression of inflammatory cytokines, enteric *Enterobacteriaceae*; ↑ TH+ neurons, enteric *Lactobacillus* and *Akkermansia*	Fang et al., 2019
Randomized placebo-controlled trial	5 male C57BL/6 mice in probiotic and saline group; 5 male C57BL/6 mice in probiotic and MPTP group; 5 male C57BL/6 mice in placebo and saline group; 5 male C57BL/6 mice in placebo and MPTP group;	Novarex^®^: 2 × 10^6^ CFU of *Lactobacillus rhamnosus* (L-GG), *Bifidobacterium animalis* (BB-12), *Lactobacillus acidophilus* (LA-5) in a commercial mixture	Once per day for 30 days	↓ MPTP-induced neurotoxicity of dopaminergic neurons, expression of MAO B and MPP^+^ in striatum, glial activation, behavioral impairments ↑ butyrate levels in brain, expression of BDNF and GDNF in SN	Srivastav et al., 2019
Randomized placebo-controlled trial	6 groups (146 male C57BL/6 mice total): placebo/saline only (*N* = 36), saline and probiotic (*N* = 32), saline and non-viable probiotic (*N* = 5), MPTP only (*N* = 36), MPTP and probiotic (*N* = 32), MPTP and non-viable probiotic (*N* = 5)	10^9^ CFUs of *Bifidobacterium breve* strain A1 [MCC1274] (*B. breve* A1)	Once per day for 4 days	↑ Hippocampal synaptic plasticity, expression of postsynaptic density protein-95 and synaptophysin, CA1 spine density; ↓ expression of neuropsin (OPN5), fear response	Ishii et al., 2021
Randomized placebo-controlled trial	6 groups (35 male Sprague-Dawley rats total): non-PD and placebo only (*N* = 5), untreated PD (*N* = 5), L-DOPA treated PD (*N* = 5), probiotic treated PD (*N* = 5), nutrient media treated PD (*N* = 5), probiotic and nutrient media treated PD (*N* = 5)	10^11^ CFUs of *Lactobacillus salivarius* subsp. *salicinius* AP-32	Once per day for 8 weeks	↓ Dopaminergic neuron loss, loss of TH+ cells in striatum and SNc, weight loss ↑ locomotor speed and stride length, mitochondrial function, antioxidative enzyme activity (GPx and SOD), fecal SCFA concentration	Nurrahma et al., 2021
Randomized placebo-controlled trial	12 adult male Sprague-Dawley rats in probiotic group; 10 adult male Sprague-Dawley rats in 6-OHDA and probiotic group; 9 adult male Sprague-Dawley rats in 6-OHDA and placebo group;	10^9^ CFUs of *Lactobacillus rhamnosus* HA-114	Once per day for 6 weeks	↓ Hippocampal-dependent cognitive deficits	Xie and Prasad, 2020
Randomized placebo-controlled trial	10 male C57BL/6 mice in MPTP group; 10 male C57BL/6 mice in MPTP and probiotic group; 10 male C57BL/6 mice in control/placebo group	5 × 10^8^ CFU of *Clostridium butyricum* strain WZMC1016	Once per day for 4 weeks	↓ Motor deficits, dopaminergic neuron loss, synaptic dysfunction, microglial activation; ↑ levels of colonic GLP-1 and GPR41/43, expression of cerebral GLP-1R	Sun et al., 2021

While these results are promising, further research is needed to evaluate both the feasibility and potential efficacy of utilizing long-term pre- and probiotic interventions. It is pertinent, however, to consider the potential importance of a neuroprotective microbiome across the lifespan to confer resilience against the development of future neurodegenerative disorders. Another important component for delaying onset and slowing progression of neurodegenerative disorders is early detection. The early identification of PD by specific subtype or phenotype would provide an opportunity for individualized treatment to target specific disease components, such as underlying dysbiosis of the gut microbiome. For example, a study of two Taiwanese PD populations, tremor and non-tremor dominant, found *Bacteroides* species to be more abundant in the non-tremor PD group, correlated with plasma levels of TNF-α and severity of motor symptoms defined by UPDRS part III, as well as an overall decreased abundance of *Prevotella* species in both groups of PD patients compared to non-PD controls (Lin et al., 2019). By evaluating the composition of gut microbiota in early stages of disease onset, it may be possible to differentiate between clinical phenotypes and therefore improve and expand upon current treatment strategies.

## Discussion and Future Perspectives

As the prevalence of PD is projected to continue to increase worldwide over the next several decades (Marras et al., 2018), it is imperative to elucidate the distinct mechanisms and pathological origins underlying this disease. There is an increased interest in understanding the potential role, if any, of the gut–brain axis in the course of disease. It is a continuously progressing field of study, which is complicated by the multitude of factors mediating the potential influence of the gut microbiome on PD including diet and lifestyle habits, levels of inflammation, presence of comorbidities, and use of supplements or medications, etc. While this avenue of research is promising, further research is needed to ascertain the role and magnitude of bidirectionality, as well as underlying mechanisms of the gut–brain axis. The evidence presented in this review suggests there may be additional pathways apart from the vagal nerve route in which α-syn aggregates may be initiated along the gut–brain axis, including immune system mediators, gut-related hormones, and microbiota-derived signaling molecules. The aggregation and propagation of enterically derived α-syn is likely indicative of an early pathological stage that may later initiate the hallmark motor and non-motor symptoms of PD. The exact role of the microbiota in α-syn-related pathology has yet to be determined, including whether enterically derived pathological α-syn is a product of early subclinical changes in brain physiology related to PD or rather deleterious shifts in the gut microbiota prior to the onset of PD pathology, or perhaps a combination of both. Further, additional research in human subjects with consistent results are needed to verify and support promising findings from murine model studies. A better understanding of the role of the microbiome–gut–brain axis in PD will inform the conception and development of novel therapeutic interventions and diagnostic tools to ultimately improve patient outcomes. Further, future research may also consider evaluating the feasibility of PD interventions targeting the gut microbiota in the context of efficacy, acceptability, and adherence.

## Author Contributions

VM and VV-M contributed to the conception and design of the manuscript. AR-Z, AS, MF, VV-M, and VM contributed to interpreting the relevant literature. AG compiled and summarized relevant literature. EK drafted and revised the manuscript. UD wrote sections of the manuscript. All authors contributed to manuscript revision, read, and approved the submitted version.

## Conflict of Interest

The authors declare that the research was conducted in the absence of any commercial or financial relationships that could be construed as a potential conflict of interest.

## Publisher’s Note

All claims expressed in this article are solely those of the authors and do not necessarily represent those of their affiliated organizations, or those of the publisher, the editors and the reviewers. Any product that may be evaluated in this article, or claim that may be made by its manufacturer, is not guaranteed or endorsed by the publisher.
